# Complete genome sequences of soil-associated *Paenibacillus polymyxa* strains ALE1, ALE2, and ALE3 generated by PacBio sequencing

**DOI:** 10.1128/mra.00183-26

**Published:** 2026-03-30

**Authors:** Thao D. Tran, SangIn Lee, Robert Hnasko, Jeffery A. McGarvey

**Affiliations:** 1 Foodborne Toxin Detection and Prevention Research Unit, Agricultural Research Service, U.S. Department of Agriculture, Albany, California, USA; 2 Produce Safety and Microbiology Research Unit, Agricultural Research Service, U.S. Department of Agriculture, Albany, California, USA; DOE Joint Genome Institute, Berkeley, California, USA

**Keywords:** *Paenibacillus*, PacBio, genome

## Abstract

*Paenibacillus polymyxa* ALE1, ALE2, and ALE3 were isolated from the soil of a lettuce farm in Salinas, California, in fall 2020. Their genomes were sequenced using the PacBio Revio platform. All three whole-genome sequences are circular; no plasmids were detected in this assembly.

## ANNOUNCEMENT

*Paenibacillus polymyxa* is a common soil bacterium that is likely an efficient plant growth-promoting and biocontrol agent (1). *P. polymyxa* ALE1, ALE2, and ALE3 were isolated from agricultural soils in Salinas, CA (its approximate center is located at coordinates 36.4357°N, 121.3653°W) as described previously (2). These were found to suppress the persistence of both *Listeria monocytogenes* and *Escherichia coli* O157:H7 using the method described by Devarajan et al. (3).

Soil between the rows of lettuce was sampled, diluted, and blended in phosphate buffer saline, and plated onto Reasoner’s 2A agar plates (for ALE1) or brain heart infusion agar plates (for ALE2 and ALE3) supplemented with cycloheximide (40 mg/L) and incubated at 25°C for 3 days. A single colony was consecutively passaged three times on tryptic soy agar (TSA) before streaking it again on TSA (Oxoid, England). A single colony was then grown overnight in tryptic soy broth at 37°C for 24 h with shaking at 200 rpm and harvested for genomic DNA extraction using sucrose-Tris with phenol-chloroform cleanup, as described previously (4). Twenty micrograms of genomic DNA was collected in total. Five micrograms of the genomic DNA was sheared using a G-tube (Covaris, MA, USA) at 3,200 × *g* in a MiniSpin plus microcentrifuge (Eppendorf, CT, USA) to target fragment lengths of 10–12 kb. The fragments were not selected in size after shearing. The SMRTbell whole-genome libraries (ALE1, ALE2, and ALE3) were prepared as instructed by the SMRTbell prep kit 3.0 procedure and checklist, barcoded using SMRTbell barcoded adapter plate 3.0 (PacBio, Menlo Park, CA, USA), and sequenced in one single-molecule real-time SMRT cell (PacBio Revio SPRQ chemistry; Chemistry v13.3.0.249,246, 300 pM on-plate loading concentration; standard HiFi library loading). The PacBio Revio run produced 12,036,608 raw reads in total (including reads of three other genomes that are not reported in this announcement). The raw data were demultiplexed and converted to HiFi read files using Microbial Genome Analysis (SMRT Link v25.2.0.266,456; PacBio, Menlo Park, CA, USA). The HiFi reads of each bacterium were used to assemble chromosomal contigs and circularized using Hifiasm 0.25.0-r726 (SCINet project of the USDA Agricultural Research Service) and confirmed using Microbial Genome Analysis (SMRT Link v25.2.0.266,456; PacBio, Menlo Park, CA, USA). Circularity was manually inspected using Geneious Prime v2026.0.2. We obtained a single circular chromosome with no additional circular contigs for each bacterium (Table 1); no plasmids were detected in this data set. Small plasmids outside the selected size range cannot be excluded. No genome rotation was performed. PacBio sequencing control (Revio SPRQ polymerase kit; PacBio, Menlo Park, CA, USA) was used as an internal sequencing control (base quality Q30 is 95.27%). The genomes were submitted to the NCBI Prokaryotic Genome Annotation Pipeline v6.10 (PGAP) for annotation (5–7). Default parameters were used for all software.

**TABLE 1:** Characteristics of the genomes of *P. polymyxa* strains ALE1, ALE2, and ALE3^*a*^

Characteristics	ALE1	ALE2	ALE3
Genome sequencing			
Total length (bp)	17,325,789,952	17,495,737,085	18,049,332,475
HiFi reads	2,098,055	2,045,760	2,400,605
HiFi read quality (median)	Q40	Q40	Q40
HiFi read length N50 (bp)	9,676	9,676	9,676
CCS analysis HiFi reads mapped (bp)	2,096,873	2,044,937	2,399,327
CCS read length N50 mapped (bp)	9,474	9,388	8,646
Contig(s)	1	1	1
Genome characteristics			
Genome size (bp)	6,069,815	5,750,723	5,812,254
Coverage (×)	2,852	3,040	3,103
GC content (%)	44.98	45.59	45.39
Circular	Yes	Yes	Yes
Coding sequences	5,499	4,838	5,074
Coding genes	5,422	4,777	5,002
Closest identified reference (% similarity)	CP178917 (88%)	CP025957 (93.4%)	CP178917 (90%)
Data availability			
GenBank accession	JBTSVW000000000	JBSYHE000000000	JBSYHF000000000
SRA accession	SRR36399346	SRR36399345	SRR36399344
BioProject	PRJNA1377667	PRJNA1377667	PRJNA1377667
BioSample	SAMN53797589	SAMN53797590	SAMN53797591

^*a*^ CCS, circular consensus sequencing; SRA, sequence read archive.

**Fig 1:**
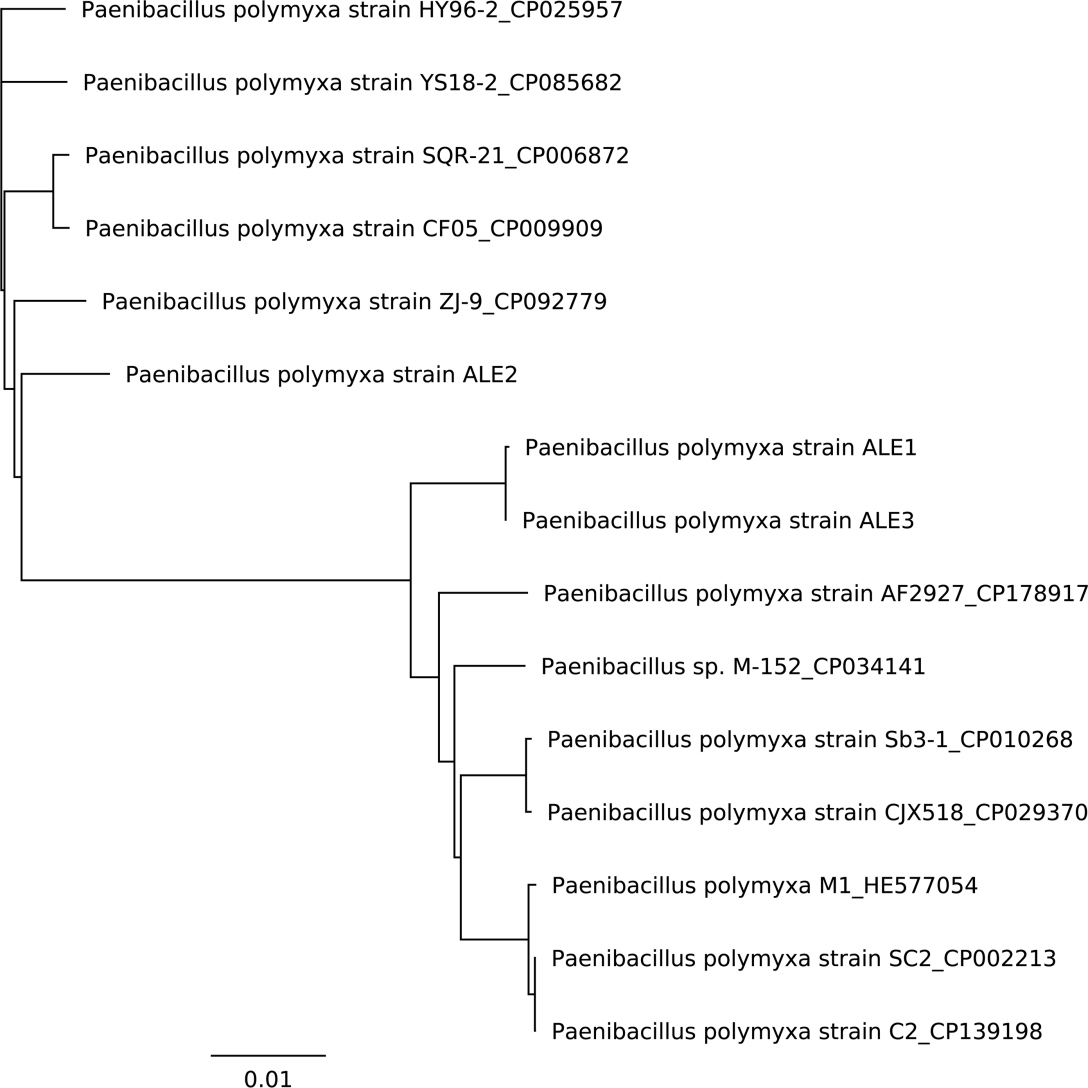
Similarities analysis between *P. polymyxa* ALE1, ALE2, and ALE3 with other *P. polymyxa* strains published on GenBank was done using the Mauve Whole Genome Aligner (Geneious Prime v2026.0.2). The phylogenetic tree was built using the distance-based neighbor-joining tree building method and the Tamura-Nei model, with bootstrapping option (number of replicates was set to 100 and the support threshold to 0) (Geneious Prime v2026.0.2). Complete bacterial genomes were imported from NCBI to Geneious as input. Default parameters were used if not described.

The characteristics of the ALE1, ALE2, and ALE3 genome assemblies are presented in Table 1. Similarity among the genome sequences of *P. polymyxa* ALE1, ALE2, and ALE3 with other GenBank-published *P. polymyxa* sequences was analyzed using Geneious v2026.0.2 and presented in Fig. 1. *P. polymyxa* ALE1, ALE2, and ALE3 genome sequences have been deposited in DDBJ/ENA/GenBank (Table 1). The HiFi raw reads for ALE1, ALE2, and ALE3 are available via sequence read archive (Table 1).

## Data Availability

This Whole Genome Shotgun project has been deposited in DDBJ/ENA/GenBank under the accession numbers shown in Table 1. The version described in this paper are the first versions JBTSVW010000000 (ALE1), JBSYHE010000000 (ALE2), and JBSYHF010000000 (ALE3).
